# Scrambler therapy for chemotherapy-induced peripheral neuropathy: A case report

**DOI:** 10.1017/S1478951525100503

**Published:** 2025-08-22

**Authors:** Zhu Wang, Michael Carducci, Giuseppe Marineo, Thomas Smith

**Affiliations:** 1 Section of Palliative Medicine, Division of General Internal Medicine, The Johns Hopkins Hospital, Baltimore, MD, USA; 2 Department of Oncology, Sidney Kimmel Comprehensive Cancer Center at Johns Hopkins, Johns Hopkins University School of Medicine, Baltimore, MD, USA; 3 Delta Research & Development, University of Rome Tor Vergata, Rome, Italy

**Keywords:** Scrambler therapy, chemotherapy-induced peripheral neuropathy, neuromodulation, oncology, case study

## Abstract

**Background:**

Chemotherapy-induced peripheral neuropathy (CIPN) is a common and debilitating side effect of cancer treatment, significantly affecting patients’ quality of life. Current pharmacological treatments are often ineffective or poorly tolerated, necessitating alternative therapeutic approaches. Scrambler Therapy (ST), a non-invasive neuromodulation technique, has shown potential for reducing neuropathic pain, but optimal dosing regimens remain undefined.

**Objective:**

This case study aims to evaluate the effectiveness of Scrambler Therapy in reducing pain levels and improving functional status in a patient with chemotherapy-induced peripheral neuropathy.

**Methods:**

A single patient diagnosed with CIPN was treated with Scrambler Therapy over a series of sessions. Pain levels and functional status were measured using standardized assessment tools before, during, and after the therapy to evaluate the impact of ST on symptom relief and daily functioning.

**Results:**

After completing the Scrambler Therapy sessions, the patient reported significant reductions in pain intensity and notable improvements in functional status. These improvements were sustained several weeks and months following the therapy, indicating the potential long-term benefits of ST for managing CIPN.

**Conclusion:**

This case study demonstrates the potential of Scrambler Therapy as an effective treatment option for reducing pain and improving functional status in patients with chemotherapy-induced peripheral neuropathy. These findings suggest that ST may provide a promising non-invasive alternative to current treatments for managing neuropathic pain in cancer patients.

## Introduction

Chemotherapy-induced peripheral neuropathy (CIPN) is one of the most common and challenging side effects of cancer treatment, affecting approximately 40–70% of patients. It manifests as pain, numbness, tingling, and weakness, significantly impairing quality of life. Moreover, CIPN can compromise cancer treatment by necessitating chemotherapy dose reductions or delays, thereby potentially reducing treatment efficacy. Currently, the American Society of Clinical Oncology guidelines do not recommend any consistently effective treatment for CIPN (Loprinzi et al. 2020). Among available therapies, duloxetine is the only drug shown to be superior to placebo, with a modest 4% reduction in pain scores (Hershman et al. 2014). However, while duloxetine may alleviate pain, it does not appear to improve numbness or tingling (Hershman et al. 2014).

Scrambler therapy (ST) is a noninvasive neuromodulation technique used to alleviate chronic pain, particularly of neuropathic origin. Unlike transcutaneous electrical nerve stimulation (TENS), which primarily targets A-beta fibers and has only temporary effects on neuropathic pain (Johnson et al. 2022), ST acts on small C-fibers. Rather than eliminating the pain signal, ST replaces it with a synthetic “non-pain” signal, effectively blocking pain perception. Although ST is FDA-cleared for safety, it remains underutilized. This is largely due to a lack of large-scale clinical trials, limited understanding of its mechanism of action, and a shortage of trained practitioners. Furthermore, challenges with insurance coverage present additional barriers to widespread adoption. To improve accessibility and integration of ST into clinical practice, more high-quality research and clinical data are urgently needed.

In the 2 largest randomized trials investigating ST for CIPN, patients experienced relief from pain, numbness, and tingling; however, symptoms typically returned within weeks of completing treatment (Loprinzi et al. 2020; Smith et al. 2020). More recently, a smaller trial reported sustained improvements in worst pain, numbness, and tingling lasting up to 6 months (Chung et al. 2024). Here, we report a case in which a patient experienced exceptionally rapid and sustained improvement in CIPN symptoms after receiving fewer than the standard 10 sessions of ST. The durability and speed of her response highlight the potential of ST to achieve meaningful clinical outcomes with a shorter treatment course. This case may encourage clinicians to further explore the utility of ST and reconsider current assumptions about treatment duration and expected outcomes in this evolving field.

## Case presentation

The patient is a woman in her 70s with a diagnosis of IgG multiple myeloma, initially arising from monoclonal gammopathy of undetermined significance in 2000. She achieved clinical remission following 4 cycles of bortezomib/dexamethasone and 2 cycles of lenalidomide/dexamethasone. In December 2020, she underwent an autologous bone marrow transplant. She currently remains on maintenance therapy with daratumumab monotherapy, having discontinued pomalidomide due to severe CIPN. Her bilateral lower extremity neuropathy has been ongoing for 6 years and was exacerbated by prior chemotherapy. She previously tried multiple therapies for her neuropathic symptoms, including gabapentin, pregabalin, nortriptyline, topical menthol, and alpha-lipoic acid – all with minimal benefit. Moderate improvement was noted with venlafaxine. At the time of her initial evaluation for ST, she reported neuropathic pain intensity of 5/10 in the right foot and 7/10 in the left foot. She ambulated with the assistance of a walker due to a slow and unsteady gait. She had been largely homebound for years, unable to stand for extended periods or take leisure walks for over a year. Her activities of daily living, such as cooking, were significantly impaired. She described feeling “stuck” at home due to the limitations imposed by her neuropathy.

## Results

**Figure 1.**
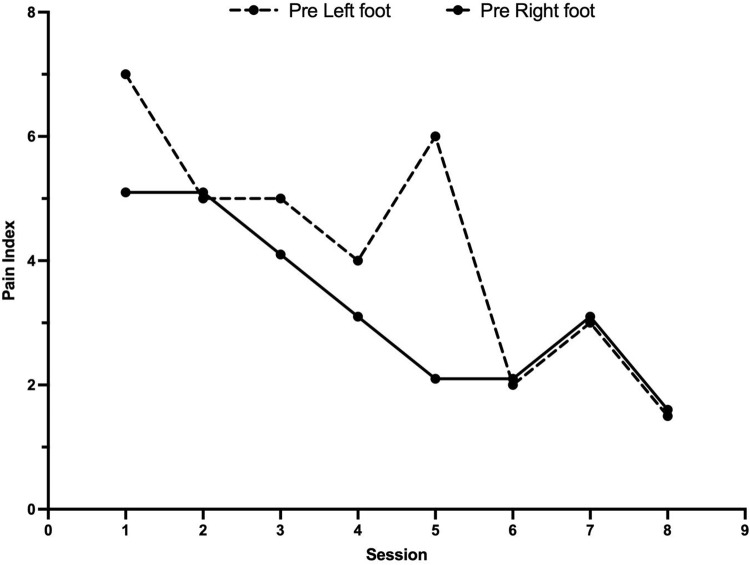
Pain score of lower extremity before and after scrambler therapy. Pre-session pain scores (0–10 scale) were recorded across eight scrambler therapy sessions for the left (dashed line) and right (solid line) foot. Both sides showed an overall reduction in baseline pain, with some mid-treatment variability. Pain was lowest at the final session, suggesting therapeutic benefit.

At her initial visit, the patient reported a significant decline in quality of life due to her CIPN, characterized by pain, numbness, and tingling rated at 5/10 in the right foot and 7/10 in the left. Following just 3 sessions of ST, her pain significantly decreased and has been sustained for over a month. Notably, she was able to take a 10-min walk which she had been unable to do for more than a year (Figure 1).

**Figure 2.**
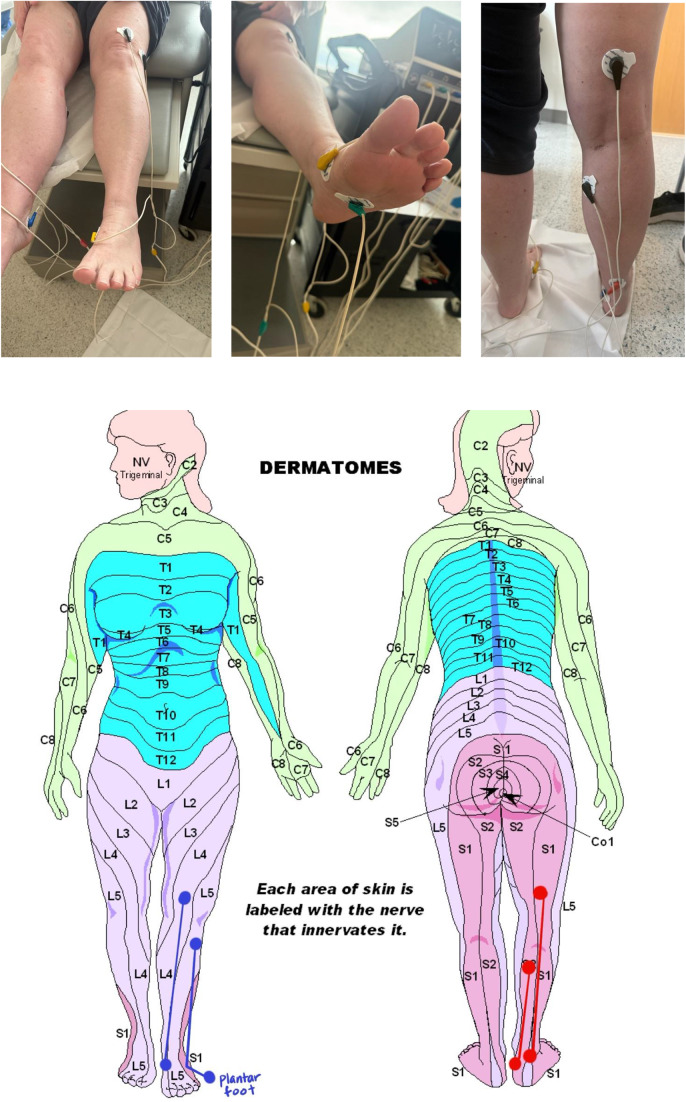
Placement of electrodes based on dermatome distribution. Dermatomal map used to guide electrode placement for scrambler therapy in a patient with bilateral foot neuropathy. Four electrode pairs were placed over the L4, L5, S1, and S2 dermatomes to target sensory pathways associated with bilateral foot pain. Dermatomal mapping was used to optimize scrambler therapy delivery

At presentation, the patient’s numbness persisted at 4/10 in the right foot and 5–6/10 in the left. By the fifth session, her numbness had decreased to 2/10 bilaterally, enabling her to resume dancing – an activity she had previously considered impossible. She was no longer reliant on a walker or any assistive device. The placement of electrodes on her lower extremities were shown in (Figure 2).

These improvements persisted through the end of her treatment. By session 8, her numbness had declined to 1/10 and remained low in the following weeks. The patient considers pain or numbness at a level of 3–4/10 to be tolerable and stated that her outcome exceeded expectations. She has become more active in her community and is now planning to travel with her daughter – something she never imagined possible prior to treatment.

## Discussion

This case illustrates a remarkable improvement in CIPN following ST, demonstrating not only rapid and near complete resolution of neuropathic pain but also substantial reduction in numbness and tingling. Importantly, these benefits persisted beyond the treatment period, suggesting that ST can offer durable symptom relief rather than merely temporary alleviation.

Notably, the patient experienced durable relief after fewer than the standard 10 ST sessions. Pain resolved entirely after just 3 sessions, and numbness steadily improved, reaching a level of 2/10 by the fifth session and stabilizing thereafter. This outcome highlights the potential for a personalized, session-to-session approach to ST dosing. This approach is consistent with the FDA-registered device instruction manual, which clearly states that treatment should be withheld if the patient returns without pain.

Despite its FDA clearance for safety, ST remains underutilized – partly due to limited awareness among clinicians and its frequent misclassification as a variant of TENS, which it is not (Smith et al. 2023). The relative novelty of ST and a lack of standardized protocols further hinder its broader adoption. Robust clinical research is urgently needed to establish evidence-based guidelines and optimize patient selection, dosing, and follow-up care.

Finally, this case raises an important question: Will the therapeutic benefits of ST persist once patients resume maintenance chemotherapy? Further investigation is warranted. We hope this case draws attention to ST’s potential as a transformative tool in managing CIPN and encourages further clinical research to better understand and harness its therapeutic impact.
